# Social capital and health consciousness based on regional differences in China: a cross-sectional study

**DOI:** 10.3389/fpubh.2025.1598121

**Published:** 2025-06-11

**Authors:** Zhen Shi, Xixi Du, Dongyang Wang, Seung Chun Paek, Patreeya Kitcharoen, Juan Li, Thammarat Marohabutr

**Affiliations:** ^1^Faculty of Social Sciences and Humanities, Mahidol University, Nakhon Pathom, Thailand; ^2^College of Humanities and Education, Inner Mongolia Medical University, Hohhot, China; ^3^Shangqiu Medicine College, Shangqiu, China; ^4^The Third People’s Hospital of Henan Province, Zhengzhou, China; ^5^School of Ethnology and Sociology, Inner Mongolia University, Hohhot, China

**Keywords:** social capital, health consciousness, regional differences, China, cross-sectional study

## Abstract

**Background:**

Health consciousness is a critical determinant of individuals’ engagement in health behaviors, while social capital influences health-related questions. This study aims to explore the relationship between social capital-comprising social participation, social trust, social networks, and social reciprocity, and health consciousness in China, with particular emphasis on regional variations.

**Methods:**

This study utilizes data from the 2021 Chinese Social Survey (CSS2021) and employs descriptive analysis, binary logistic regression, and subsample regression to examine the effects of social capital on health consciousness, with a focus on regional differences across China.

**Results:**

There are significant regional differences in health consciousness among Chinese residents. Overall, the proportion of residents exhibiting a high level of health consciousness accounted for more than half (67.3%), with eastern China leading at 68.8% and the western region at a lower level of 64.3%. Social participation, social trust, and social reciprocity were found to significantly influence health consciousness. Regionally, the eastern region was influenced by social participation, social trust, and social networks; the central region by social participation alone; and the western region by social reciprocity (*p* < 0.05). In terms of socio-demographic and economic characteristics, the eastern region’s health consciousness was significantly influenced by age, gender, education, family economic status, and happiness. In the central region, factors included gender, marital status, education, family economic status, recent medical experience, and social equity cognition. In the western region, marital status, education, income group, family economic status, and social insurance satisfaction were significant factors (*p* < 0.05).

**Conclusion:**

This study highlights significant regional disparities in health consciousness among Chinese residents with variations closely linked to socioeconomic development. Social capital, including social participation, social trust, social networks, and social reciprocity, plays a crucial role, with its impact varying across regions. Factors such as age, gender, education, income, and family economic status influence health consciousness differently depending on the region. The findings underscore the need for region-specific health policies that address socioeconomic factors and strengthen social capital, aiming to improve health consciousness and public health outcomes across China.

## 1 Introduction

Health consciousness, defined as the awareness and concern for one’s physical and mental well-being, reflects the specific concern about one’s own health status and symbolizes a conscious treatment of one’s own health (1). This construct encompasses both cognitive awareness and behavioral engagement with health-promoting activities. Especially, the COVID-19 pandemic has significantly elevated health consciousness as a critical focus in global public health discourse (2). This global crisis has heightened awareness of health risks (3), spurred reflections on physical well-being (4), and driven the widespread adoption of health-promoting behaviors, including the “home fitness trend” (5). As a result, individuals have developed a deeper appreciation for the importance of health, fostering lasting behavioral changes aimed at safeguarding personal well-being (6–8). As such, health consciousness is considered a pivotal determinant of health-related behaviors and outcomes, playing a significant role in shaping public health interventions and policies.

Health consciousness is influenced by a broad array of factors, which can be broadly categorized into sociodemographic, economic, and cultural dimensions. Key sociodemographic factors—such as gender, age, region, ethnicity, and marital status—have been shown to shape individual attitudes toward health (5–11). Additionally, socioeconomic factors such as education level, income, and occupation are critical in determining one’s access to health information, healthcare services, and resources for adopting healthier lifestyles (4, 12–21). Furthermore, emerging evidence suggests that cultural norms and regional disparities can also significantly influence health consciousness (22).

Social capital is a multifaceted construct encompassing resources embedded within social structures, mobilized by individuals or groups to achieve their goals (23, 24). It comprises social networks, social trust, social reciprocity, and social participation, which significantly influence health behaviors and outcomes. Social networks link individuals or groups through relational ties, while social trust reflects confidence in others, including strangers. Social reciprocity emphasizes mutual benefits in social interactions, while social participation involves active community engagement, such as volunteering or joining organizations (25–27). Social capital positively affects self-rated health, happiness, and physical and mental well-being (28–30), as well as life satisfaction among populations in different countries (31–33). Community involvement and robust social networks notably enhance scientific health consciousness (9, 34, 35), serving as a conduit between socioeconomic status and health consciousness (36).

In China, the vast territorial expanse is marked by significant regional disparities. Factors such as economic development, healthcare infrastructure, environmental conditions, and socio-cultural influences all contribute to these disparities, particularly between the eastern, central, and western regions. Eastern China generally enjoys higher levels of income, better access to healthcare services, and improved living conditions (37). In contrast, the central and western regions often face economic challenges that limit their ability to invest in healthcare infrastructure and public health initiatives (38). Studies have shown that certain health-seeking behaviors and preventive practices are more prevalent in urban areas than in rural settings (39). Additionally, ethnic minorities in the western region may face additional barriers due to language differences and cultural beliefs (40).

Significant regional disparities in the eastern, central, and western China profoundly impact population health. These disparities stem from uneven socioeconomic development, varying public health infrastructure, and distinct cultural attitudes toward health, leading to divergent levels of health consciousness across regions. The COVID-19 pandemic has further reshaped public perceptions of health, exacerbating existing inequalities in health consciousness. Despite the well-established link between social capital and health outcomes, critical research gaps remain. Notably, the influence of regional differences on the relationship between social capital and health consciousness is underexplored, particularly in the Chinese context, where economic, cultural, and healthcare variations are pronounced. Furthermore, social capital is often treated as a single construct, overlooking the potentially unique contributions of its distinct dimensions, such as trust, reciprocity, and social participation, to health consciousness.

This study seeks to investigate the relationship between social capital and health consciousness in China, with a focus on regional differences. By examining how social participation, social trust, social networks, and social reciprocity shape health consciousness across different regions, the study aims to shed light on the critical role that social capital plays in fostering or hindering health consciousness. The findings from this study will contribute to a deeper understanding of the regional nuances that influence public health behavior and will inform more targeted, context-specific health promotion strategies.

## 2 Methods

### 2.1 Data sources

The data used in this study were taken from the data of 2021 Chinese Social Survey (CSS2021). These data constitute a nationwide large-scale continuous sample survey project initiated by the Institute of Sociology of Chinese Academy of Social Sciences. Data concerning China’s social changes in the transitional period through long-term longitudinal surveys on Chinese residents’ employment, family and social life, social attitudes, and other aspects to provide detailed and scientific basic information for social science research and government decision-making. The survey adopts the method of multistage mixed probability sampling. The survey subjects are Chinese residents aged 18–69 years old. The survey contents include the aspects of the employment, family and social life, and social attitudes of Chinese residents. The CSS2021 sampled 604 village (residential) committees under the jurisdiction of 151 counties (cities and districts) in 31 provinces, municipalities, and autonomous regions of China (excluding Hong Kong, Macao and Taiwan). The total sample size was 10,136. The CSS2021 survey included two versions, Form A and Form B. Form A, which included the health consciousness variable, contained 5,119 samples. The main exclusion criterion was the presence of missing values in some independent variables selected for this study. After removing missing values, the final sample size, which included health consciousness, was 4,083.

### 2.2 Variable selection

#### 2.2.1 Dependent variable

##### 2.2.1.1 Health consciousness

The variables of health consciousness in this study focus on examining people’s attention to health-related dimensions which are composed of questions about six aspects. The question is “Since the COVID-19 pandemic, have you experienced any changes in the following aspects?,” the answers include: “Pay attention to physical exercise and health protection,” “Pay attention to the mental health of oneself and family members,” “Pay attention to family and affection,” “Be willing to trust the kindness of strangers,” “Believe that China will get better and better,” and “Pay attention to ecology and environmental protection.” The responses are “Increased/Enhanced,” “No change,” “Decreased/Weakened,” and “Uncertain.” In this study, the choice of “Increase/Enhanced” is set as “Improved” with an assigned value of 1. All other cases are set as “No improvement” with an assigned value of 0. Health consciousness is the sum of the above six aspects ranging from 0 to 6. The Cronbach’s alpha for this scale was 0.771. The measurement of health consciousness varies globally, with some scholars employing standardized scales to assess this construct, although these scales are not widely utilized (41–44). Others have opted to capture health consciousness through a range of related dimensions, each emphasizing distinct aspects (4, 11). Drawing on prior research, this study therefore adopts the six aspects to measure health consciousness. This approach not only enhances measurement validity but also offers a more practical and accessible means of conducting surveys across diverse populations. Referring to previous studies (11, 45), health consciousness is typically divided into high and low categories based on the median value. Respondents with scores higher than 3 typically exhibit more positive health behaviors or higher health consciousness, while those with scores lower than 3 may demonstrate lower levels of health consciousness. In this study, the score of health consciousness of 3 points or less is set as “Low,” and the score of 4 points or more is set as “High.”

#### 2.2.2 Independent variables

With reference to previous studies (46, 47), this study divides social capital variables into four dimensions: social participation, social trust, social networks, and social reciprocity.

##### 2.2.2.1 Social participation

From the question posed to respondents in the questionnaire is: “In the last 2 years, have you participated in any of the following activities?” The multiple choice answers include: (1) discussing political issues with others or online friends; (2) reflecting social problems to newspapers, radio stations, internet forums, and other media; (3) reflecting opinions to government departments (including by telephone, e-mail etc.); (4) participating in public policy and public affairs demonstration meetings with professional knowledge; (5) expressing personal opinions on government policies through various channels; (6) attending public policy hearings organized by government departments; (7) petitioning government departments; (8) participating in major decision-making discussions in the village/unit; (9) participating in community or self-organized social welfare activities such as voluntary blood donation, voluntary environmental cleaning and voluntary help for the older adults, disabled, and sick; (10) participating in religious activities; (11) participating in online/offline collective rights protection actions; and (12) none of above. In this study, the choice of any public affairs participation denotes “Participation” with an assigned value of 1. The choice “12. “None of above” denotes “No participation” with an assigned value of 0.

##### 2.2.2.2 Social trust

Based on the question from the questionnaire “On a scale of 1–10, please express your assessment of the current level of trust between people, with 1 being very untrusting and 10 being very trusting,” the social trust variable in this study is a continuous variable. Higher respondents’ score denotes higher level of social trust.

##### 2.2.2.3 Social networks

The questions from the questionnaire include: “I feel very good relationship with the people in my circle/group” and “My circle/group makes me feel safe and secure,” with the corresponding answers: “Strongly disagree,” “Do not agree,” “Hard to say,” “Somewhat agree” and, “Strongly agree” with values ranged 1 to 5, respectively. The social network variable of this study is a summary of the two questions. Social networks are a continuous variable with assigned scores between 2 and 10. Higher score of social networks signifies better degree of social networks.

##### 2.2.2.4 Social reciprocity

The questions from the questionnaire include: “People who do good deeds in society do not expect to be rewarded” and “I believe that most people in society are kind,” with the corresponding answers: “Strongly disagree,” “Do not agree,” “Hard to say,” “Somewhat agree,” and “Strongly agree” with a rating of 1 to 5, respectively. The social reciprocity variable in this study is a summary of the two questions. Social reciprocity is a continuous variable with a score ranging from 2 to 10. Higher social reciprocity score signifies higher degree of social reciprocity.

#### 2.2.3 Control variables

The control variables include sociodemographic factors, socioeconomic factors, and social behavioral and cognitive factors.

The sociodemographic factors include age, gender, marital status, household status, and health insurance status. In this study, age is divided into three groups: 0 = Youth, 1 = Middle-aged adults, and 2 = Older adults. Gender is categorized into two groups: 0 = Female and 1 = Male. Marital status is categorized into 0 = No spouse and 1 = Have a spouse. Household is categorized into 0 = Rural and 1 = Urban. Health insurance status is coded as 0 = None and 1 = Have.

The socioeconomic factors include education level, family economic status, and income group. Education level is assigned 0 = Primary school and below, 1 = Junior high school, 2 = High school (technical secondary school), and 3 = Junior college or above. Family economic status is coded as 0 = Not getting better and 1 = Getting better. Income group is divided into 0 = Low-income group, 1 = Middle-income group and 2 = High-income group. To be clear, with reference to the general classification of middle-income groups in China (48, 49), this study defines people whose annual personal income (26,400 yuan/year in the survey data) is equal to or less than 0.75 times of the median value (that is, less than 19,800 yuan/year) as the low-income group. Those whose annual personal income is higher than 0.75 times of the median value but less than or equal to 2 times of the median value (that is, the annual income is 19,800–52,800 yuan) are defined as the middle-income group. Those whose annual personal income is more than 2 times of the median value (that is, greater than 52,800 yuan/year) are defined as the high-income group.

The social behavioral and cognitive factors include recent medical experience, happiness, social insurance satisfaction, and social equity cognition. Recent medical experience is categorized into 0 = None and 1 = Yes. Happiness, social insurance satisfaction, and social equity cognition are divided into two groups 0 = Low and 1 = High.

### 2.3 Statistical methods

The overall characteristics of the respondents were described as percentages for categorical variables. Between-group comparisons were conducted using the *χ*^2^ test for categorical variables. Additionally, *χ*^2^ tests were applied to examine regional differences in the distributions of sociodemographic factors, socioeconomic factors, social behavioral and cognitive factors, social capital, and health consciousness. Binary logistic regression analysis was employed to investigate the association between social capital and health consciousness, with results expressed as odds ratios (OR) and 95% confidence intervals (CI). All statistical analyses were performed using SPSS version 22.0, with a significance threshold set at *p* = 0.05.

## 3 Results

**Figure 1:**
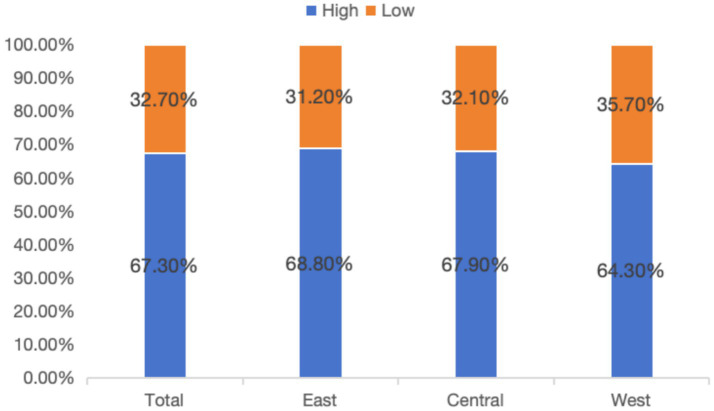
Differences in health consciousness at overall level and across different regions.

In the post-COVID-19 era, there are significant regional differences in health consciousness among Chinese residents. Overall, the proportion of residents exhibiting high level of health consciousness accounted for more than half (67.3%). Regionally, residents in eastern China exhibit the highest proportion of high health consciousness (68.8%). The proportion of residents in the central region with high health consciousness is 67.9%, while the western region demonstrates a relatively lower proportion (64.3%), falling below the national average (67.3%). These results are shown in Figure 1 on the differences in health consciousness at overall level and across different regions. From the perspective of individual characteristics, health consciousness levels vary significantly across age, household status, education level, family economic status, income level, happiness, social insurance satisfaction, and social equity cognition, both at the national level and within different regions. Specifically, youth, urban residents, individuals with an education level of junior college or above, those whose family economic conditions have improved over the past 5 years, higher-income groups, and residents with high levels of happiness, social insurance satisfaction, and social equity cognition tend to exhibit higher levels of health consciousness.

In terms of age, there is a significant disparity in health consciousness levels across different ages in the eastern region. Specifically, the proportion of youth with high health consciousness is 21.2% higher than that of the older adults. In contrast, while the proportion of youth with high health consciousness also exceeds that of the older adults in the central region, the gap narrows to only 13%. Regarding urban–rural differences, the western region exhibits a pronounced disparity in health consciousness levels between urban and rural residents, whereas the eastern region shows only a minor difference, with urban residents having a slightly higher proportion (1.8%) of high health consciousness compared to their rural counterparts. Health consciousness levels among Chinese residents vary significantly by educational attainment, particularly in the central and western regions. Concerning family economic status and income level, the largest gap is observed in the western region. The central region demonstrates the most substantial disparity in happiness and social security satisfaction. Lastly, regarding perceptions of social equity cognition, the western region again shows the widest gap. These results are shown in Table 1 on the descriptive analysis of the differences in health consciousness and personal characteristics at overall level and across different regions.

**Table 1:** Descriptive analysis of the differences in health consciousness and personal characteristics at overall level and across different regions.

Characteristics		Total		East		Central		West		Total	
		Health consciousness		Health consciousness		Health consciousness		Health consciousness		*X* ^2^	*p*-value
		Low	High	Low	High	Low	High	Low	High		
Total		1,336 (32.7)	2,747 (67.3)	537 (31.2)	1,183 (68.8)	401 (32.1)	848 (67.9)	398 (35.7)	716 (64.3)		
Age	Youth	239 (26.0)	682 (74.0)	94 (23.7)	303 (76.3)	74 (26.1)	209 (73.9)	71 (29.5)	170 (70.5)	72.911	<0.001
	Middle-aged	682 (30.9)	1,526 (69.1)	255 (28.2)	649 (71.8)	218 (31.7)	469 (68.3)	209 (33.9)	408 (66.1)		
	Older adults	415 (43.5)	539 (56.5)	188 (44.9)	231 (55.1)	109 (39.1)	170 (60.9)	118 (46.1)	138 (53.9)		
Gender	Female	679 (31.6)	1,473 (68.4)	273 (29.4)	657 (70.6)	203 (30.5)	462 (69.5)	203 (36.4)	354 (63.6)	2.825	0.093
	Male	657 (34.0)	1,274 (66)	264 (33.4)	526 (66.6)	198 (33.9)	386 (66.1)	195 (35.0)	362 (65.0)		
Marital status	No spouse	272 (34.8)	509 (65.2)	97 (30.3)	223 (69.7)	89 (35.9)	159 (64.1)	86 (40.4)	127 (59.6)	1.946	0.163
	Have a spouse	1,064 (32.2)	2,238 (67.8)	440 (31.4)	960 (68.6)	312 (31.2)	689 (68.8)	312 (34.6)	589 (65.4)		
Household status	Rural	894 (35.1)	1,651 (64.9)	316 (32.0)	671 (68.0)	280 (34.6)	530 (65.4)	298 (39.8)	450 (60.2)	17.776	<0.001
	Urban	442 (28.7)	1,096 (71.3)	221 (30.2)	512 (69.8)	121 (27.6)	318 (72.4)	100 (27.3)	266 (72.7)		
Health insurance status	None	502 (34.9)	936 (65.1)	199 (33.3)	398 (66.7)	161 (34.8)	301 (65.2)	142 (37.5)	237 (62.5)	4.83	0.028
	Have	834 (31.5)	1811 (68.5)	338 (30.1)	785 (69.9)	240 (30.5)	547 (69.5)	256 (34.8)	479 (65.2)		
Education level	Primary school and below	549 (46.2)	639 (53.8)	186 (45.4)	224 (54.6)	157 (43.7)	202 (56.3)	206 (49.2)	213 (50.8)	148.745	<0.001
	Junior high school	380 (29.5)	910 (70.5)	150 (27.2)	401 (72.8)	131 (31.3)	287 (68.7)	99 (30.8)	222 (69.2)		
	High school (technical secondary school)	216 (28.0)	555 (72.0)	104 (29.0)	255 (71.0)	67 (27.9)	173 (72.1)	45 (26.2)	127 (73.8)		
	Junior college or above	191 (22.9)	643 (77.1)	97 (24.3)	303 (75.8)	46 (19.8)	186 (80.2)	48 (23.8)	154 (76.2)		
Family economic status	Not getting better	592 (41.0)	851 (59.0)	255 (38.8)	403 (61.2)	170 (40.0)	255 (60.0)	167 (46.4)	193 (53.6)	69.915	<0.001
	Getting better	744 (28.2)	1896 (71.8)	282 (26.6)	780 (73.4)	231 (28.0)	593 (72.0)	231 (30.6)	523 (69.4)		
Income level	Low-income group	616 (39.3)	952 (60.7)	196 (36.4)	342 (63.6)	188 (36.9)	321 (63.1)	232 (44.5)	289 (55.5)	50.096	<0.001
	Middle-income group	469 (29.0)	1,149 (71.0)	203 (29.2)	492 (70.8)	152 (28.8)	375 (71.2)	114 (28.8)	282 (71.2)		
	High-income group	251 (28.0)	646 (72.0)	138 (28.3)	349 (71.7)	61 (28.6)	152 (71.4)	52 (26.4)	145 (73.6)		
Recent medical experience	None	590 (33.5)	1,170 (66.5)	232 (31.4)	507 (68.6)	192 (34.1)	371 (65.9)	166 (36.2)	292 (63.8)	0.903	0.342
	Yes	746 (32.1)	1,577 (67.9)	305 (31.1)	676 (68.9)	209 (30.5)	477 (69.5)	232 (35.4)	424 (64.6)		
Happiness	Low	195 (50.9)	188 (49.1)	93 (50.8)	90 (49.2)	58 (53.2)	51 (46.8)	44 (48.4)	47 (51.6)	63.543	<0.001
	High	1,141 (30.8)	2,559 (69.2)	444 (28.9)	1,093 (71.1)	343 (30.1)	797 (69.9)	354 (34.6)	669 (65.4)		
Social insurance satisfaction	Low	483 (44.9)	593 (55.1)	184 (41.8)	256 (58.2)	167 (45.6)	199 (54.4)	132 (48.9)	138 (51.1)	98.254	<0.001
	High	853 (28.4)	2,154 (71.6)	353 (27.6)	927 (72.4)	234 (26.5)	649 (73.5)	266 (31.5)	578 (68.5)		
Social equity cognition	Low	408 (44.4)	510 (55.6)	154 (40.8)	223 (59.2)	137 (43.5)	178 (56.5)	117 (51.8)	109 (48.2)	73.935	<0.001
	High	928 (29.3)	2,237 (70.7)	383 (28.5)	960 (71.5)	264 (28.3)	670 (71.7)	281 (31.6)	607 (68.4)		

A binary logistic regression model was employed to examine the relationship between the four dimensions of social capital and health consciousness among Chinese residents. This study revealed that social participation, social trust, and social reciprocity had significant impacts on residents’ health consciousness (*p* < 0.05). Residents who participated in social activities exhibited 1.332 times higher health consciousness compared to those who did not participate. Moreover, higher levels of social networks and social reciprocity were associated with increased levels of health consciousness.

In addition, socio-demographic and economic characteristics, including age, gender, marital status, education level, income group, family economic status, recent medical experiences, happiness level, social insurance satisfaction, and social equity cognition, were found to significantly influence the residents’ health consciousness (*p* < 0.05). Table 2 shows the logistic regression of the social capital and health consciousness.

**Table 2:** Overall binary logistic regression of social capital and health consciousness.

Variables	Total		
	*B*	OR (95% CI)	*p*-value
Social participation (ref: no)	0.287	1.332 (1.140–1.556)	<0.001
Social trust	0.044	1.045 (1.007–1.085)	0.019
Social networks	0.029	1.029 (0.977–1.084)	0.274
Social reciprocity	0.063	1.065 (1.017–1.116)	0.008
Control variables	Controlled		
Nagelkerke *R*^2^	0.126		
Constant	−2.056	0.128	<0.001
Observations	4,083		

Subsample binary logistic regression analysis was conducted to explore the relationship between social capital and health consciousness among residents in different regions. The findings indicate that in the eastern region, social participation, social trust, and social networks significantly influenced residents’ health consciousness (*p* < 0.05). Higher level of social networks and social reciprocity demotes higher level of health consciousness of residents. The health consciousness of residents with social participation was 1.477 times higher than that of residents without social participation. In the eastern region, social participation is the most significant factor affecting the residents’ health consciousness. At the same time, it is known that, compared with the overall situation, the social trust dimension of social capital does not have a significant impact on the health consciousness of residents in the eastern region. However, the social networks had no significant effect on the overall situation, but had significant effect on the health consciousness of residents in the eastern region (*p* > 0.05). In the central region, only social participation was found to have a significant impact on health consciousness (*p* < 0.05). The health consciousness of residents with social participation was 1.385 times higher than that of residents without social participation. The study did not find that social networks, social trust, and social reciprocity had significant effects on health consciousness of residents in the central region (*p* > 0.05). In the western region, social reciprocity emerged as the sole dimension of social capital significantly affecting residents’ health consciousness (*p* < 0.05). Higher level of social reciprocity signifies higher level of residents’ health consciousness. However, the study did not find that social participation, social networks and social trust had significant effects on the health consciousness of residents in the western region (*p* > 0.05).

Regarding the socio-demographic and economic characteristics, in the eastern region, age, gender, education level, family economic status, and happiness level significantly influenced health consciousness. In the central region, gender, marital status, education level, family economic status, recent medical experience, and social equity cognition were identified as significant factors. In the western region, marital status, education level, income group, family economic status, and social insurance satisfaction were significantly associated with health consciousness (*p* < 0.05). Table 3 shows the subsample binary logistic regression of the social capital and the health consciousness in different regions.

**Table 3:** Binary logistic regression of social capital and health consciousness in different regions.

Variables	East		Central		West	
	*B*	OR (95% CI)	*B*	OR (95% CI)	*B*	OR (95% CI)
Social participation (ref: no)	0.390^**^	1.477 (1.143–1.907)	0.326^*^	1.385 (1.037–1.849)	0.266	1.305 (0.984–1.730)
Social trust	0.079^*^	1.082 (1.016–1.152)	0.055	1.057 (0.990–1.129)	−0.004	0.997 (0.931–1.066)
Social networks	0.090^*^	1.094 (1.006–1.190)	0.027	1.027 (0.933–1.131)	−0.036	0.965 (0.877–1.060)
Social reciprocity	0.033	1.034 (0.958–1.115)	0.025	1.025 (0.942–1.115)	0.156^*^	1.168 (1.068–1.278)
Control variables	Controlled		Controlled		Controlled	
Nagelkerke *R*^2^	1.133		0.129		0.168	
Constant	−1.920^***^	0.147	−2.007^***^	0.134	−2.666^***^	0.070
Observations	1720		1,249		1,114	

*p < 0.05, **p < 0.01, ***p < 0.001.

## 4 Discussion

There are obvious differences in socioeconomic status and regional differences in health consciousness among Chinese residents. Especially, in China, residents in the eastern region exhibit the highest level of health consciousness, followed by those in the central region, while the western region residents show the lowest level of health consciousness. The regional disparities in health consciousness among Chinese residents are consistent with the economic and social development conditions across the eastern, central, and western regions of China (50).

The health consciousness of Chinese residents is influenced by a variety of factors. From the socio-demographic and economic perspectives, older adults tend to exhibit lower levels of health consciousness, with the older adults showing particularly lower health consciousness compared to the youth. Additionally, males generally display lower health consciousness than females. Residents with a spouse tend to have significantly higher health consciousness than those without. Furthermore, individuals with higher levels of education also demonstrate higher health consciousness. The middle-income group shows significantly higher health consciousness compared to the low-income group. Residents with improved family economic status and those with recent medical experiences tend to have notably higher levels of health consciousness. Moreover, residents who report higher happiness level, social insurance satisfaction, and a stronger sense of social equity cognition tend to exhibit significantly higher health consciousness.

These factors exert varying effects on the health consciousness of residents across different regions in China. Gender has a significant impact on the health consciousness of residents in the eastern and central regions only. Marital status, on the other hand, significantly affects the health consciousness of residents in the central and western regions. Age and happiness levels have a notable effect on the health consciousness of residents in the eastern region alone. Recent medical experience and social equity cognition significantly influence the health consciousness of residents in the central region. Finally, income group and social insurance satisfaction have a significant effect on the health consciousness of residents in the western region. Both education level and family economic status significantly influence health consciousness across the three regions. Higher education correlates with better economic status and enhanced health consciousness. Education improves individuals’ ability to process and apply health information (51), while greater educational attainment is linked to higher health literacy, enabling individuals to better understand and analyze health-related information (52). Improved economic conditions increase life satisfaction and mental well-being, and provide broader access to health information, which fosters greater attention to personal and family health management (53–55). Moreover, individuals with higher education typically have better employment opportunities and greater financial resources, allowing them to access more health services and preventive measures. These advantages further facilitate their ability to prioritize and maintain health (56). China exhibits significant regional disparities in socioeconomic development. In general, the eastern region boasts a more developed economy, higher income levels, better educational attainment, and more extensive healthcare resources, along with a more diverse range of social capital forms. In addition, the modernized cultural orientation in the east enables residents to more readily adopt new health concepts, collectively enhancing their health consciousness. In contrast, the central and western regions, characterized by lower initial levels of economic, educational, and healthcare investment, often have community networks and cultural practices that are less aligned with public health promotion, resulting in comparatively weaker health consciousness (57–59). Particularly in the digital era, the eastern region leads in adopting digital health tools, which significantly enhance health consciousness and self-management. On the contrary, the western region lags behind due to limited internet penetration and lower level of digital literacy (60).

This study, while controlling for socio-demographic and economic factors, focuses on analyzing the impact of social capital on the residents’ health consciousness and its regional variations. Overall, this study found that social capital, including social participation, social trust, and social reciprocity, significantly influences the health consciousness of Chinese residents. No significant impact was found regarding social networks. However, owing to its cross-sectional design, the analysis cannot establish causal relationships between these constructs or assess changes over time, thereby limiting inferences about the directionality or temporal dynamics of the observed associations. Nevertheless, it could provide some theoretical interpretations of these associations. The potential reason for this is that social participation, on the one hand, promotes the sharing of information among residents, including health lectures and exercise promotion, making it easier for individuals to access health-related knowledge (61). On the other hand, social participation enhances residents’ sense of social connection and belonging, contributing to emotional support and mental well-being, which fosters a positive attitude toward health management (62). Social trust, in turn, reduces the likelihood of residents being influenced by false health information, allowing them to more easily obtain accurate and authoritative health knowledge. It also facilitates the spread of health information between individuals, thereby contributing to the formation of higher health consciousness (63). Social reciprocity emphasizes mutual support and cooperation among community members. On the one hand, residents can promote adherence to health behaviors through mutual supervision and reminders (64). On the other hand, residents are more willing to engage in the sharing and utilization of health resources, which enhances their health knowledge and consciousness (65).

The impact of social capital on the health consciousness of residents in different regions of China shows considerable regional variation. Social participation, social trust, and social networks significantly affect the health consciousness of residents in the eastern region, whereas social reciprocity, which affects health consciousness for the general population, does not show a significant effect in the eastern region. Furthermore, social networks, which do not have a significant impact on the general population, have a significant positive impact on the health consciousness of residents in the eastern region. This may be because the eastern region of China has a large floating population and is economically more developed. Developed social networks in this region enhance the dissemination of information and social support, thus improving health consciousness (66).

In the central region, the residents’ health consciousness is significantly influenced by social participation, while in the western region, social reciprocity has a significant impact on health consciousness. Social trust and social reciprocity, which affect general population, do not significantly affect the health consciousness of residents in the central region. Social participation and social trust, which significantly influence the general population, do not show a significant effect on the health consciousness of residents in the western region. The central region of China is undergoing rapid economic development and accelerated urbanization, which has gradually increased residents’ social participation. Participation in community activities, volunteer services, and other social practices provides residents with important opportunities to access health knowledge and receive health education (5). Nevertheless, social reciprocity emphasizes mutual assistance and resource sharing between individuals (27). In the western region, social reciprocity plays a more prominent role because public resources and social services are relatively low. Residents in this region are more dependent on reciprocal relationships for daily life and health management. Neighborly assistance and community support have become key channels for residents to obtain health information and behavioral guidance (67).

## 5 Limitation

This study has several limitations. First, the CSS2021 data used in this research is cross-sectional, collected after the onset of the COVID-19 pandemic in 2021. While respondents were asked to subjectively assess whether their health consciousness had changed due to the pandemic, the absence of pre-pandemic data prevents direct comparisons, making it difficult to evaluate shifts in health consciousness over time. Moreover, the reliance on self-reported data introduces the potential for various biases, particularly social desirability bias, where respondents may overestimate positive behaviors or attitudes related to social capital and health consciousness. This form of bias can lead to inflated associations and may limit the validity of the findings. Given the cross-sectional nature of this study, it is not possible to establish causal relationships between the variables or capture trends over time. Although this design is useful for identifying potential associations, it inherently limits the ability to infer the directionality or causality of the observed relationships between social capital and health consciousness. Health consciousness is a multifaceted concept with various interpretations and measurement methods. Scholars have developed different scales to capture its diverse dimensions. This study focuses on a psychologically oriented measurement of health consciousness, which contrasts with behaviorally oriented approaches. Future research should strive to develop a standardized, universally accepted measurement scale for health consciousness, which would enable more robust and comparable studies across different populations and contexts.

## 6 Conclusion

This study highlights substantial regional disparities in health consciousness among Chinese residents, with significant differences observed across the eastern, central, and western regions. These variations are linked to differing levels of economic and social development. Key socio-demographic and economic factors, including age, gender, marital status, education, income, and family economic status, significantly influence health consciousness, with their impact varying by region. Additionally, the social capital factors such as social participation, social trust, and social reciprocity play a critical role, with context-specific effects shaped by regional characteristics.

These findings underscore the need for region-specific public health strategies that address both socioeconomic and social capital determinants. In the eastern region, efforts should focus on developing community-based programs that foster mutual support, build supportive social networks, and enhance social reciprocity. In the central region, promoting active community participation should be emphasized, while in the western region, interventions should aim at strengthening local-level reciprocity to improve health consciousness. This study offers practical insights for policymakers seeking to reduce regional health disparities and improve population health.

## Data Availability

The datasets presented in this study can be found in online repositories. The names of the repository/repositories and accession number(s) can be found in the article/Supplementary material.
