# Supplementary material for: Social capital and health consciousness based on regional differences in China: a cross-sectional study
Source: Front Public Health. 2025 Jun 11;13:1598121. doi: 10.3389/fpubh.2025.1598121 (PMC12187719; doi:10.3389/fpubh.2025.1598121)
Supplement: Supplementary file 1 [file Supplementary_file_1.docx]

**
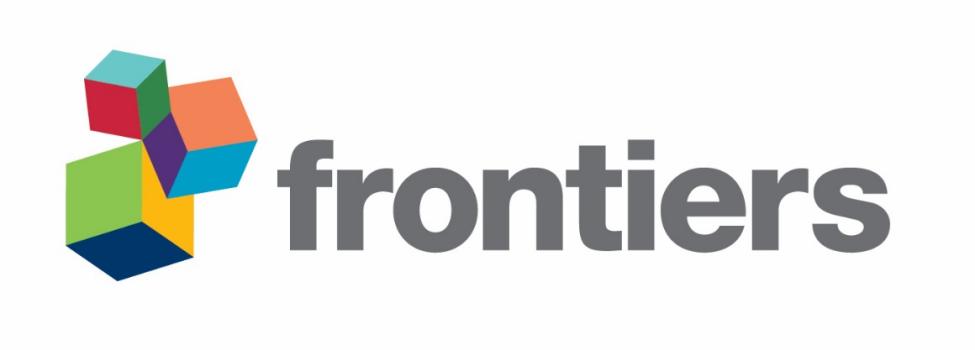
**

**Supplementary Table 1 Overall binary logistic regression of social capital and health consciousness.**

| **Variables** | **Total** | | |
| --- | --- | --- | --- |
|  | **B** | **OR (95% CI)** | **P-value** |
| Social participation (ref: No) | 0.287 | 1.332 (1.140-1.556) | < 0.001 |
| Social trust | 0.044 | 1.045 (1.007-1.085) | 0.019 |
| Social networks | 0.029 | 1.029 (0.977-1.084) | 0.274 |
| Social reciprocity | 0.063 | 1.065 (1.017-1.116) | 0.008 |
| Age (ref: Youth) |  |  |  |
| Middle-aged | - 0.016 | 0.984 (0.801-1.209) | 0.876 |
| Older adults | - 0.467 | 0.627 (0.494-0.795) | < 0.001 |
| Gender (ref: Female) | - 0.223 | 0.800 (0.693-0.923) | 0.002 |
| Marital status (ref: No spouse) | 0.218 | 1.244 (1.029-1.503) | 0.024 |
| Household status (ref: Rural) | 0.001 | 1.001 (0.849-1.179) | 0.995 |
| Health insurance status (ref: None) | 0.039 | 1.040 (0.899-1.201) | 0.600 |
| Education level（ref: Primary school and below） |  |  |  |
| Junior high school | 0.658 | 1.930 (1.608-2.317) | < 0.001 |
| High school (technical secondary school) | 0.720 | 2.054 (1.641-2.570) | < 0.001 |
| Junior college or above | 0.848 | 2.335 (1.796-3.037) | < 0.001 |
| Income group (ref: Low-income group) |  |  |  |
| Middle-income group | 0.196 | 1.217 (1.030-1.438) | 0.021 |
| High-income group | - 0.021 | 0.979 (0.789-1.216) | 0.849 |
| Family economic status (ref: Not getting better) | 0.400 | 1.492 (1.291-1.724) | < 0.001 |
| Recent medical experience (ref: None) | 0.203 | 1.225 (1.063-1.411) | 0.005 |
| Happiness level (ref: Low) | 0.378 | 1.459 (1.149-1.853) | 0.002 |
| Social insurance satisfaction (ref: Low) | 0.219 | 1.245 (1.038-1.494) | 0.018 |
| Social equity cognition (ref: Low) | 0.315 | 1.371 (1.153-1.629) | < 0.001 |
| Nagelkerke R2 | 0.126 | | |
| Constant | - 2.056 | 0.128 | < 0.001 |
| Observations | 4083 | | |

**Supplementary Table 2 Overall binary logistic regression of social capital and health consciousness.**

| **Variables** | **East** | | | **Central** | | | **West** | | |
| --- | --- | --- | --- | --- | --- | --- | --- | --- | --- |
|  | **B** | **OR (95% CI)** | **P-value** | **B** | **OR (95% CI)** | **P-value** | **B** | **OR (95% CI)** | **P-value** |
| Social participation (ref: No) | 0.390 | 1.477 (1.143-1.907) | 0.003 | 0.326 | 1.385 (1.037-1.849) | 0.027 | 0.266 | 1.305 (0.984-1.730) | 0.065 |
| Social trust | 0.079 | 1.082 (1.016-1.152) | 0.014 | 0.055 | 1.057 (0.990-1.129) | 0.099 | -0.004 | 0.997 (0.931-1.066) | 0.919 |
| Social networks | 0.090 | 1.094 (1.006-1.190) | 0.035 | 0.027 | 1.027 (0.933-1.131) | 0.589 | -0.036 | 0.965 (0.877-1.060) | 0.455 |
| Social reciprocity | 0.033 | 1.034 (0.958-1.115) | 0.390 | 0.025 | 1.025 (0.942-1.115) | 0.570 | 0.156 | 1.168 (1.068-1.278) | 0.001 |
| Age (ref: Youth) |  |  |  |  |  |  |  |  |  |
| Middle-aged | -0.058 | 0.944 (0.688-1.295) | 0.720 | -0.053 | 0.948 (0.639-1.408) | 0.793 | 0.074 | 1.077 (0.723-1.605) | 0.714 |
| Older adults | -0.736 | 0.479 (0.332-0.690) | <0.001 | -0.232 | 0.793 (0.502-1.253) | 0.321 | -0.401 | 0.669 (0.424-1.058) | 0.086 |
| Gender (ref: Female) | -0.257 | 0.773(0.616-0.970) | 0.026 | -0.309 | 0.734 (0.562-0.959) | 0.023 | -0.073 | 0.930 (0.708-1.221) | 0.601 |
| Marital status (ref: No spouse) | -0.080 | 0.923 (0.681-1.250) | 0.604 | 0.443 | 1.557 (1.103-2.198) | 0.012 | 0.441 | 1.554 (1.084-2.228) | 0.016 |
| Household status (ref: Rural) | -0.057 | 0.945 (0.738-1.211) | 0.654 | -0.032 | 0.968 (0.713-1.315) | 0.836 | 0.124 | 1.132 (0.812-1.578) | 0.463 |
| Health insurance status (ref: None) | 0.025 | 1.025 (0.814-1.291) | 0.835 | 0.114 | 1.121 (0.863-1.455) | 0.392 | 0.062 | 1.064 (0.804-1.407) | 0.666 |
| Education level (ref: Primary school and below) |  |  |  |  |  |  |  |  |  |
| Junior high school | 0.734 | 2.084 (1.541-2.818) | < 0.001 | 0.479 | 1.614 (1.163-2.241) | 0.004 | 0.702 | 2.017 (1.431-2.843) | < 0.001 |
| High school (technical secondary school) | 0.571 | 1.771 (1.255-2.499) | 0.001 | 0.686 | 1.986 (1.312-3.006) | 0.001 | 0.950 | 2.585 (1.634-4.091) | < 0.001 |
| Junior college or above | 0.638 | 1.893 (1.275-2.811) | 0.002 | 1.027 | 2.791 (1.673-4.658) | < 0.001 | 0.908 | 2.479 (1.476-4.166) | 0.001 |
| Income group (ref: Low-income group) |  |  |  |  |  |  |  |  |  |
| Middle-income group | 0.111 | 1.117 (0.852-1.465) | 0.423 | 0.126 | 1.135 (0.837-1.539) | 0.416 | 0.331 | 1.393 (1.015-1.911) | 0.040 |
| High-income group | -0.058 | 0.944 (0.681-1.308) | 0.729 | -0.219 | 0.804 (0.526-1.227) | 0.311 | 0.124 | 1.132 (0.727-1.763) | 0.584 |
| Family economic status (ref: Not getting better) | 0.358 | 1.431 (1.142-1.793) | 0.002 | 0.354 | 1.424 (1.091-1.859) | 0.009 | 0.576 | 1.779 (1.342-2.358) | < 0.001 |
| Recent medical experience (ref: None) | 0.183 | 1.201 (0.960-1.504) | 0.110 | 0.295 | 1.343 (1.037-1.738) | 0.025 | 0.169 | 1.184 (0.903-1.554) | 0.222 |
| Happiness level (ref: Low) | 0.519 | 1.681 (1.170-2.414) | 0.005 | 0.408 | 1.504 (0.966-2.342) | 0.071 | 0.133 | 1.142 (0.705-1.852) | 0.589 |
| Social insurance satisfaction (ref: Low) | -0.001 | 0.999 (0.741-1.347) | 0.995 | 0.205 | 1.228 (0.897-1.680) | 0.199 | 0.564 | 1.757 (1.226-2.518) | 0.002 |
| Social equity cognition (ref: Low) | 0.236 | 1.266 (0.958-1.674) | 0.097 | 0.446 | 1.562 (1.156-2.111) | 0.004 | 0.280 | 1.324 (0.942-1.861) | 0.107 |
| Nagelkerke R2 | 1.133 | | | 0.129 | | | 0.168 | | |
| Constant | -1.920 | 0.147 | < 0.001 | -2.007 | 0.134 | < 0.001 | -2.666 | 0.070 | < 0.001 |
| Observations | 1720 | | | 1249 | | | 1114 | | |
